# PLCZ1 gene mutation leads to fertilization disorder: a case report

**DOI:** 10.5935/1518-0557.20250028

**Published:** 2025

**Authors:** Yuxing Xiong, Yan Liu, Mei Tang, Sha Shi, Yu Wang

**Affiliations:** 1 Department of Reproductive Medicine, Puren Hospital Affiliated to Wuhan University of Science and Technology,Wuhan, China; 2 School of Medicine, Medical Department, Wuhan University of Science and Technology, Wuhan, China

**Keywords:** fertilization disorders, *PLCZ1* gene, intracytoplasmic sperm injection, assisted oocyte activation

## Abstract

**Objective:** Sperm-specific phospholipase C-zeta (*PLCζ*) is a sperm-derived oocyte activating factor, which can induce Ca^2+^ oscillation and initiate oocyte activation. The mutation of this gene will affect oocyte activation and lead to fertilization failure(FF). In this paper, we report a fertilization disorder caused by a PLCZ1 gene mutation. The patient’s peripheral blood was collected for whole exon detection. The results showed that the patient had heterozygous mutations in *PLCZ1* gene c.1733 C > T (p.M578L) and c.471 G > C (p.M157I), and the male’s brother also carried heterozygous mutations in the gene. Finally, the patient obtained clinical pregnancy by in vitro fertilization with donor sperm. At the same time, the related literature on *PLCZ1* gene mutation at home and abroad was reviewed and analyzed to improve the clinicians’ understanding of the *PLCZ1* gene and fertilization disorders.

## INTRODUCTION

Infertility is a global reproductive health problem. More than 50 million couples worldwide suffer from infertility. Assisted reproductive technology (ART) has brought new hope to patients, but there are still some unexplained fertilization disorders.

The causes of fertilization disorders are complex and diverse. Oocyte activation defect (OAD) is widely regarded as the primary reason for fertilization failure. Intracytoplasmic sperm injection (ICSI) is commonly employed to enhance fertilization rates following in vitro fertilization (IVF) failure. However, fertilization failure (FF) still occurs in 1-5% of ICSI cycles. Artificial oocyte activation (AOA) is a technique for artificially increasing intracellular calcium. ICSI in conjunction with AOA (ICSI-AOA) enhances fertilization rates in patients with male factor-related oocyte activation issues. Genetic influences significantly contribute to oocyte activation issues, potentially resulting in fertilization failure post-ICSI-AOA. The *PLCZ1* gene is the earliest discovered pathogenic gene that causes male factor fertilization failure. It is an oocyte activator that initiates oocyte activation by inducing Ca^2+^ oscillations. In this study, whole exome sequencing was utilized to identify the genetic factors behind recurrent fertilization failure following ICSI-AOA in a family with non-consanguineous marriage. We identified novel compound heterozygous mutations c.1733 C > T (p.M578L) and c.471 G > C (p.M157I) in the PLCZ1 gene of a male patient.

## CASE PRESENTATION

The patient, a female, 33 years old, was admitted to the reproductive center in May 2021 due to 12 years of infertility without contraception. Usually irregular menstruation, cycle 5-7 days / 30-60 days, BMI: 19.33kg / m^2^. Gynecologic examination: vulva was normal, the vagina was smooth, the uterus was horizontal, the cervix was smooth and soft, no tenderness, mass was not touched in the double attachment area, no tenderness. Female basal endocrine: FSH: 7.98mIU / ml, LH: 7.38mIU / ml, E2: 27.94pg / ml, AMH: 4.97ng / ml, AFC: about 20. Hysterosalpingography in other hospitals showed that the uterine cavity was normal and bilateral fallopian tubes were blocked. The karyotype is 46, XX. In 2012, due to ‘endometrial polyps’, hysteroscopic endometrial lesion resection was performed in other hospitals, and bilateral fallopian tube plastic surgery was performed due to ‘bilateral fallopian tube obstruction’. The male was 32 years old, BMI: 23.74 kg / m^2^, no abnormality was found in the semen routine, and the chromosome karyotype was 46, XY. Both sides had no bad habits and were not engaged in reproductive toxicity-related work. The parents were healthy, and his brother had been infertile for many years, denying any familial history of infectious or hereditary diseases.

In 2017, IVF assisted pregnancy treatment was performed in other hospitals. In the first cycle, a long-acting protocol program was performed, and 14 oocytes were collected. No fertilization, no transferable embryos. In the second cycle, the antagonist regimen was performed, and 2 oocytes were collected. ICSI assisted pregnancy treatment was performed without fertilization, and the specific embryo situation was unknown.

The first cycle of our hospital in May 2021: natural luteal phase: Gn was used for a total of 8 days, trigger day E2:5524pg / mL, P:9.23ng / mL. Oocytes were collected 36 hours after the trigger, and 13 MII oocytes were collected. On the first day following ICSI, two 2PN were normally fertilized; on the second day, one each of 3PN, 4PN, and multinucleate were seen to form; on the third day, one 3PN and two 4PN were seen; and on the third day of normal fertilization, the embryos were graded 8-cell grade II and were not transferred in the fresh cycle. In July 2021, two Day3 embryos were transferred in the resuscitation cycle without pregnancy. In combination with the male brother’s infertility for many years and assisted reproductive IVF infertility, communication with patients is recommended to consider whole exon gene detection, and the next treatment is performed after the cause is clarified. The patient refused and asked for ICSI treatment again.

The second cycle of our hospital in April 2022: the antagonist regimen was used, Gn was used for a total of 8 days, E2:3107pg / ml, P:0.61ng / ml on the trigger day. At 36 h after the trigger, 24 oocytes were collected, including 21 MII, 1 MI and 2 GV. The patient failed in one IVF and two ICSI. For patients with low fertilization or no fertilization, AOA can significantly improve the fertilization rate and the success rate of embryonic development (Ferrer-Buitrago *et al.*, 2018)in particular, regarding its impact on assisted reproduction technology (ART. As a result, the patient received AOA therapy. One 2PN was normally fertilized, one 2PN was observed on the second day, two unequally divided cells, two whole fragments, and one 3PN was observed on the third day. To continue to observe the embryo potential, a free blastocyst culture was performed for the patient, and 5 embryos were degraded. Finally, one Day3 available embryo was formed, and no pregnancy was transferred in the fresh cycle.

**Figure 1:**
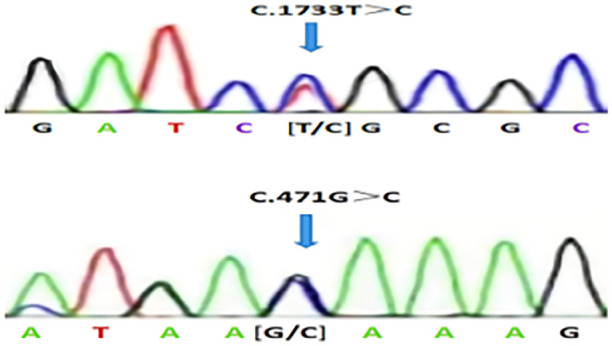
Localization of mutations in the PLCZ1 gene.

No clinical pregnancy was found in 1 IVF and 3 ICSI cycles. The MII oocytes obtained in each cycle were ideal, the oocyte maturity was acceptable, and the possibility of sperm abnormality was considered. AOA intervention has been carried out, but fertilization is still poor, and 3PN and 4PN have occurred many times, considering whether there are genetic abnormalities in oocytes or sperm. In May 2023, the patient returned to the hospital again, and the couple along with the male brother and his wife performed whole exon gene detection together. The male gene detection found heterozygous mutations in PL0D1 and *PLCZ1* genes, and the clinical significance was unknown (Table 1, Figure 1). The male brother gene indicated a heterozygous mutation of *PLCZ1* at the same site, and the clinical significance was unknown (Table 2). The MSHS gene heterozygous mutation was suspected to be pathogenic, and the female gene was not abnormal. Combined with the patient’s medical history and appeal, after discussion by the ethics committee, the patient chose to provide sperm for pregnancy. Comprehensive consideration, the woman’s ovarian function is better, bilateral fallopian tubes are unobstructed, and the patient does not get pregnant after 2 cycles of artificial insemination in other hospitals.

**Table 1:** Male exons tested for PLOD1, PLCZ1 mutation sites.

Gene Name	Chromosome location	Nucleic acid changes	Amino acid change	Exon Introns	Heterozygosity	Diseases and genetic patterns	Pathogenicity
PLOD1	chr1:12027051	c.1658C>T	p.P553L	Exon 16	Hybridization	Ehlers-Danlos syndrome type 6, autosomal recessive inheritance	Clinical significance unclear
PLCZ1	chr12:18837072	c.1733T>C	p.M578T	Exon 14	Hybridization	Type 17 spermatogenic dysfunction, autosomal recessive inheritance	Clinical significance unclear
PLCZ1	chr12:18837072	c.471G>C	p.M157I	Exon 5	Hybridization	Type 17 spermatogenic dysfunction, autosomal recessive inheritance	Clinical significance unclear

**Table 2:** Exons of male brother to detect MSH5, PLCZ1 mutation sites.

Gene Name	Chromosome location	Nucleic acid changes	Amino acid change	Exon Introns	Heterozygosity	Diseases and genetic patterns	Pathogenicity
MSH5	chr6:g.31727290G	c.1495+1G>T	NA	INTRO	Hybridization	Premature ovarian failure type 13, autosomal recessive inheritance	Suspected to be pathogenic
PLCZ1	chr12:g.18837072A>G	c.1733T>C	p.M578T	Exon 14	Hybridization	Type 17 spermatogenic dysfunction, autosomal recessive inheritance	Clinical significance unclear
PLCZ1	chr12:g.18872463C>G	c.471G>C	p.M157I	Exon 5	Hybridization	Type 17 spermatogenic dysfunction, autosomal recessive inheritance	Clinical significance unclear

In May 2023, our hospital underwent donor IVF treatment, a short-acting long-term regimen, Gn was used for a total of 9 days, trigger day E2:8285pg / ml, P:0.88ng / ml. Thirty-six hours after the trigger, 13 oocytes were collected. Eleven 2PN were formed after in vitro fertilization of the donor sperm and two were unfertilized. The patient required all blastocysts to be cultured to form 6 blastocysts. The resuscitation cycle was started in July 2023, and two Day5 embryos were transplanted. The Serum human chorionic gonadotropin (HCG) was 328.1U / L on the 15^th^ day after operation, and the HCG was 4490U / L on the 24^th^ day after operation. B-ultrasound showed gestational sac and cardiac tube pulsation, which was determined as clinical pregnancy. By the time of writing this article, the patient had had a full-term cesarean section of a male live baby, now healthy, nearly one year old.

## DISCUSSION

In this study, a novel PLCZ1 compound heterozygous mutation was found in an infertile male who experienced repeated fertilization failure even after ICSI-AOA by whole exome sequencing.

*PLCZ1* is a sperm-specific protein and an important factor for oocyte activation. It is mainly located in the perinuclear sheath between the inner membrane and the nuclear membrane of the sperm head (Che *et al.*, 2024)the gene encoding PLCζ, cause male infertility and intracytoplasmic sperm injection (ICSI. After sperm-oocyte plasma membrane fusion, the entry of *PLCZ1* into oocytes can trigger the release of Ca^2+^ in the endoplasmic reticulum, and the continuous fluctuation of Ca^2+^ concentration induces the release of oocyte block and initiates embryonic development. The autosomal recessive mutation of the *PLCZ1* gene is associated with type 17 spermatogenesis dysfunction, and its mutation or deletion affects oocyte activation, resulting in a low fertilization rate or complete fertilization failure (Jones *et al.*, 2022). Kashir *et al.* (2012)with abnormal testicular gene expression considered to be a major cause. Certain types of male infertility are caused by failure of the sperm to activate the oocyte, a process normally regulated by calcium oscillations, thought to be induced by a sperm-specific phospholipase C, PLCzeta (PLCζ first discovered the first heterozygous mutation of the *PLCZ1* gene in infertile men diagnosed with oocyte activation defects and complete fertilization failure. In recent years, related cases of *PLCZ1* gene mutation have been reported. Yuan *et al.* (2020)there are still many unexplained aspects of FF. Here, we aimed to assess the clinical and genetic characteristics of two families experiencing primary infertility with FF.\nMETHODS: We have characterized two families from China. All of the infertile couples presented with similar clinical phenotypes, that is, partial or total fertilization failure in repeated cycles. We performed Sanger sequencing of their WEE2, TLE6, and PLCZ1 genes, and further bioinformatics and functional analyses were performed to identify the pathogenic elements of the variants.\nRESULTS: We identified novel compound heterozygous mutations c.1259C>T (p.P420L found new compound heterozygous mutations c.1259C > T (p.P420L) and c.1727T > C (p.L576P) in the *PLCZ1* gene in a male patient with complete fertilization failure. Lin *et al.* (2023) found a compound heterozygous mutation of the *PLCZ1* gene (p.C196X, p.c 403_404del) and a homozygous mutation of p.W536X in 2 patients with fertilization failure.

In this instance, the patient had a history of infertility for 12 years. After excluding other infertility factors, one cycle of IVF and three cycles of ICSI were performed, and the fertilization of the patient was not ideal. It was highly suspected that the patient’s husband and wife had genetic defects. In order to find out the reason, the patient’s husband and wife were subjected to whole exon detection. The results suggested that the man had a *PLCZ1* heterozygous mutation with PLOD1 mutation. The first mutation site of *PLCZ1* was c.1733C > T (p.M578L), and the second mutation site was c.471G > C (p.M157I), which was a new mutation site. *PLCZ1* gene heterozygous mutation also exists in the same locus in the male brother. *PLCZ1* has become an important gene that can not be ignored in the genetic screening and diagnosis of infertile men.

Although several pathogenic *PLCZ1* gene mutation types have been found, there is still a lack of effective treatment. ICSI injects a single sperm into the cytoplasm of MII oocytes through a micromanipulation system to fertilize them, which helps to avoid most male infertility (Palermo *et al.*, 2017). Although ICSI technology can improve the fertilization rate after IVF failure, there are still 1%-5% of challenging infertile men who have total fertilization failure (TFF) or low fertilization rate (fertilization rate < 30 %) after ICSI treatment, which is all in the category of fertilization disorders (Yeste *et al.*, 2016). For patients with complete fertilization failure caused by *PLCZ1* mutation, the AOA method is recommended for intervention. AOA can significantly improve the embryo fertilization rate of patients by increasing the level of calcium ions in oocytes to assist oocyte activation. It has been reported that ICSI combined with AOA is effective for infertile men with *PLCZ1* mutations, which can partially rescue fertilization failure and contribute to successful pregnancy (Torra-Massana *et al.*, 2019; Antonova *et al.*, 2024)mainly due to oocyte activation failure (OAF. In this case, we found that not all patients could benefit from AOA even if fertilization failed under ICSI and AOA treatment (Nicholson *et al.*, 2024)Dundee between January 2014 and December 2021 (n = 231. Wang *et al.* (2020)the infertile man with PLCZ1 mutation was treated with intracytoplasmic sperm injection (ICSI identified a homozygous *PLCZ1* nonsense mutation c.588C > A (p.Cys196Ter), which had a low fertilization rate and poor embryo development after ICSI + AOA treatment. Consequently, it becomes clear that PLCZ1 is necessary for early embryonic development and fertilization.

## CONCLUSION

In this paper, the patient finally obtained clinical pregnancy by in vitro fertilization with donor sperm. The causes of fertilization disorders are complex and diverse, which is a difficult problem for clinicians. A new PLCZ1 mutation site, c.471G > C (p.M157I), was discovered in a pair of brothers with a fertilization abnormality. This finding broadened the spectrum of PLCZ1 gene mutations associated with male infertility and further supported the crucial function PLCZ1 plays in human fertilization. For patients with primary infertility and multiple assisted reproductive failure, especially those with low fertilization rate or repeated fertilization failure, it is recommended to carry out genetic testing as soon as possible to clarify the genetic etiology. For patients with genetic problems in one of the spouses, if they have brothers and sisters, they should also improve the genetic testing of the family as soon as possible before giving birth, so as to achieve accurate diagnosis and treatment, improve the pregnancy outcome of patients, and avoid repeated fertilization failure to patients. Unnecessary economic losses and physical and mental injuries.
